# A systematic review and meta-analysis of physical environmental enrichment to improve animal welfare-related outcomes in indoor cattle

**DOI:** 10.1017/awf.2025.28

**Published:** 2025-06-13

**Authors:** Ganimet Unsal, Kate F Johnson, Sokratis Stergiadis, Richard Bennett, Zoe E Barker

**Affiliations:** School of Agriculture, Policy and Development, University of Reading, Reading RG6 6EU, UK

**Keywords:** animal welfare, calves, dairy cows, environmental enrichment, indoor housing, physical enrichment, well-being

## Abstract

This systematic review aims to evaluate the effectiveness of various physical environmental enrichment items such as brushes, ropes, teats, chains, balls, cowhides/blocks, at improving the welfare of indoor-housed calves, heifers, and cattle. This review of 33 peer-reviewed papers and one industry report evaluated different welfare-related outcomes following physical environmental enrichment, including feed intake, lying time, play and exploratory behaviour, aggression, stereotypic behaviour and cross-sucking behaviour. The results of the meta-analysis revealed that calves and heifers enrolled in experimental studies using enrichment items had significantly improved growth rates, and increased locomotor play, but the overall reduction in cross-sucking behaviour was small and non-significant. The effect of enrichment on feed intake, aggression/stereotypic behaviour, play behaviour, cleanliness score contrasted between studies, with some reporting improvements while others showed no effect of environmental enrichment in these parameters. The risk of bias assessment revealed limitations in researcher blinding, sequence generation, and allocation concealment across the literature assessing the effectiveness of environmental enrichment on animal welfare. Overall, this review underscores the significant positive impact of physical enrichment on the welfare and behaviour of indoor-housed cattle, while highlighting the need for further research to optimise enrichment strategies across different cattle age groups and housing conditions.

## Introduction

Indoor housing systems for dairy cattle, which may include free-stall barns, loose yards and tie-stall systems, have several advantages, like controlling the environment, protecting animals from detrimental weather conditions, and facilitating management practices such as controlled feeding, monitoring of health status, and easy access to animals for medical treatment (Mandel *et al.* 2016). Nevertheless, indoor housing can also present welfare challenges. For instance, reduced opportunities for physical activity, limited space, and intensified management practices can alter social dynamics and potentially increase negative interactions, especially around feeding (Arnott *et al.* 2017). Constrained environments may also reduce cows’ autonomy over who they spend time near, heightening the risk of conflict (Chopra *et al.* 2020). Additionally, indoor environments may elevate the risk of disease transmission (Fraser *et al.* 2013). Specifically in dairy cows and calves, these adverse effects can lead to behavioural and performance-related issues, such as reduced feeding and rumination, increased aggression and stereotypies, and lower growth rates (Mason *et al.* 2007; von Keyserlingk *et al.* 2009). Although measures such as growth rate, feed intake, and cleanliness are not direct indicators of welfare states, they can indirectly influence health, comfort, and overall well-being, making them relevant for understanding how housing and management conditions affect welfare-related outcomes.

There are several strategies proposed for addressing the negative effects on animal welfare in indoor housing systems, including modifying light and temperature controls, enhancing diet plans, and providing physical environmental enrichment (Liebenberg 2017; van de Weerd & Ison 2019). Environmental enrichment, in particular, can provide mental and physical stimulation to animals and promote the expression of natural behaviours (Ellingsen *et al.* 2014) as well as increasing physical activity, and reducing stereotypical behaviours (Smith & Taylor 1996; Beattie *et al.* 2000; Young 2013).

The use of physical environmental enrichment is now a widely accepted method for improving the welfare of indoor-housed livestock, particularly for laying hens, broilers, and pigs (Mandel *et al.* 2016; Orihuela *et al.* 2018; Pedersen *et al.* 2020; Padilha-Boaretto *et al.* 2021). This is reflected in the recent updates to the Codes of Practice for laying hens and pigs in the UK (DEFRA 2018, 2020). While the Brambell Committee’s original Freedoms (Brambell 1965) formed the foundation for modern welfare standards, the Farm Animal Welfare Council later refined and expanded them into what are now widely recognised as the Five Freedoms (FAWC 2009). These freedoms emphasise enabling animals to exhibit natural behaviours, ensuring physical comfort, and facilitating healthy social interactions. More recent frameworks, such as the Five Domains model, encompass not only the animal’s physical condition, diet and environmental interactions, but also behaviour, and mental state, with emphasis being placed on the animal being able to experience positive welfare states (Mellor 2016). This is especially relevant regarding the use of environmental enrichments such as balls, brushes, chains, ropes, and toys. While guidelines exist for certain species, their direct application and the supporting evidence for dairy cattle remain limited. For example, while the recent Codes of Practice address the welfare of laying hens and pigs (DEFRA 2018, 2020), they provide less detailed guidance on the needs of dairy cattle, highlighting a gap in practice. Indeed, recent comprehensive reviews, such as the 2023 EFSA calf welfare review (EFSA et al. 2023), have highlighted the importance of environmental enrichment for calf welfare. These reviews identify specific areas where further research and more detailed recommendations are needed, reinforcing the value of a thorough evaluation of enrichment strategies for dairy cattle. This underscores the need for a thorough review to evaluate the relevance and impact of enrichment on dairy cattle welfare, including indirect measures related to performance and hygiene.

This systematic review and meta-analysis aims to assess the available evidence on the effectiveness of physical environmental enrichment in indoor farm management, particularly focusing on calves, replacement heifers, and lactating cows. This study attempts to address the existing literature gap by comprehensively describing and assessing different strategies. The overall aim will be achieved via the following specific objectives: (1) summarising the current knowledge on the use of physical environmental enrichment in indoor cattle husbandry; (2) evaluating how different physical enrichment types influence welfare-related outcomes, including both behavioural measures and indirectly related performance and cleanliness indicators; and (3) identifying factors that may affect the success of environmental enrichment programmes in indoor dairy farm management.

## Materials and methods

This systematic review included a comprehensive search of the scientific literature on physical environmental enrichment in indoor cattle husbandry, specifically calves, heifers, and dairy cows. The search was carried out using a predetermined search strategy and includes four bibliographic databases and grey literature sources. The inclusion and exclusion criteria of the review were based on the Preferred Reporting Items for Systematic Reviews and Meta-Analyses (PRISMA) guidelines (Page *et* *al.* 2021). Included studies were evaluated using SYRCLE’s Risk of Bias tool (Hooijmans *et al.* 2014). Data from the included studies were analysed using the narrative synthesis method and meta-analysis. Ethical approval was not required for this study since it is a systematic review, consisting of meta-analysis of previously published research, and no new animal experiments.

### Search strategy

A systematic search of the scientific literature was conducted to find studies on the use of environmental enrichment in indoor livestock. The following databases were used for the search: Web of Science, Scopus, CAB Abstracts, and PubMed. There are no start date restrictions, while the end date for the literature review was 15th January 2023.

The search keywords for this systematic review were selected to ensure a comprehensive and focused exploration of the available literature, guided by the PI(C)O framework — Population (P), Intervention (I), Comparison (C), and Outcome (O). The Population element was addressed by incorporating keywords ("cattle" OR “cow” OR “calf” OR “calves” OR “heifer”) AND ("indoor" OR “shelter” OR “stall” OR “barn”) to specifically target indoor-housed cattle. For the Intervention element, keywords including ("enrichment" OR “toy” OR “device” OR “material” OR “enriched” OR “physical” OR “environmental”) were used to capture the various types of enrichment tools being evaluated. We did not explicitly define the Comparison element in the search terms to allow for a broader inclusion of studies, including single-group or observational designs that might not specify a ‘control’ in their indexing. Nonetheless, Table 1 lists potential comparators (e.g. no enrichment) to reflect how enrichment and non-enrichment conditions could be assessed once eligible studies were located. The Outcome element encompasses any behavioural or performance study, where performance studies (e.g. growth rates) serve as indirect indicators of welfare, effectiveness of enrichment (e.g. reduction in stress-related behaviours), and efficiency of enrichment (cost-effectiveness and practicality of enrichment interventions). We did not define Outcome in the search terms to allow for a broad scoping review of various welfare indicators. Additionally, reference lists of included studies were manually searched to identify any relevant articles that may not have been captured through the initial keyword search.

**Table 1.** PICO Framework: the inclusion and exclusion criteria of physical environmental enrichment to improve animal welfare-related outcomes in indoor cattle for the systematic review

PICO	Inclusion Criteria	Exclusion Criteria
Population	Calves All developmental stages (neonatal, weaning) Heifers All stages (bulling, pregnant) Cows All production types and stages (beef, dairy, in milk, dry)	
Intervention	Physical environmental enrichment items Indoor housing Individual or group housing	Other types of enrichment Feedlot farming Free range
Comparison (If included i.e. treatment control trials)	No physical environmental enrichment items Indoor housing Individual or group housing	Other types of enrichment Feedlot farming Free range
Outcomes	Any behavioural or performance study Effectiveness of enrichment Efficiency of enrichment	Lack of detail – qualitative descriptions without any quantitative data

### Inclusion criteria and study selection

The search included studies published in English that report on the effects of physical environmental enrichment on a range of outcome parameters including, but not limited to, growth rate, mortality, morbidity, and behavioural changes. Table 1 shows the inclusion and exclusion criteria that were closely followed at each step of the process. Peer-reviewed publications that reported original research on cattle of any breed, age, or sex, where environmental enrichment was a key aspect of the study were included. Although the search primarily targeted peer-reviewed studies, we did not exclude grey literature. For example, if an industry report found through reference lists met the inclusion criteria, it was included. Studies were excluded if they did not provide sufficient detail on the methodology, specifically, clear descriptions of the environmental enrichment items used, the controls in place, sample sizes, duration of the study, and the variables measured. If a study did not focus primarily on enrichment intervention or lacked these details, it was not included.

**Figure 1.**
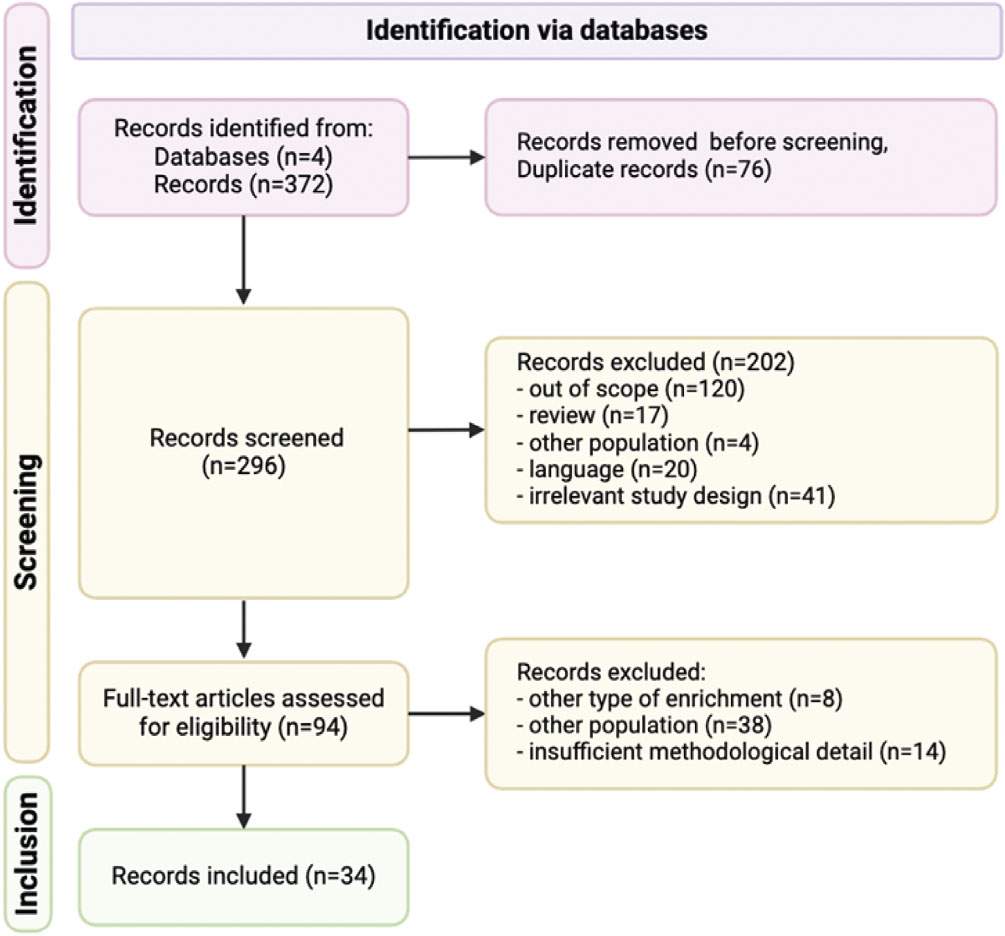
Illustration of the PRISMA guidelines (Page *et al.* 2021) search, screening and inclusion process for a systematic review of physical environmental enrichment to improve animal welfare-related outcomes in indoor cattle. The number of articles identified from Web of Science, Scopus, CAB Abstracts, and PubMed, and numbers removed at each screening stage are reported in parentheses.

The selection of studies was completed by GU. Any uncertainties were resolved through discussion with the wider review team. The selection process was carried out in three steps: (1) scanning of the title and abstract; (2) reviewing full-text; and (3) inclusion in the review. This process is illustrated in Figure 1.

### Risk of bias assessment

**Figure 2.**
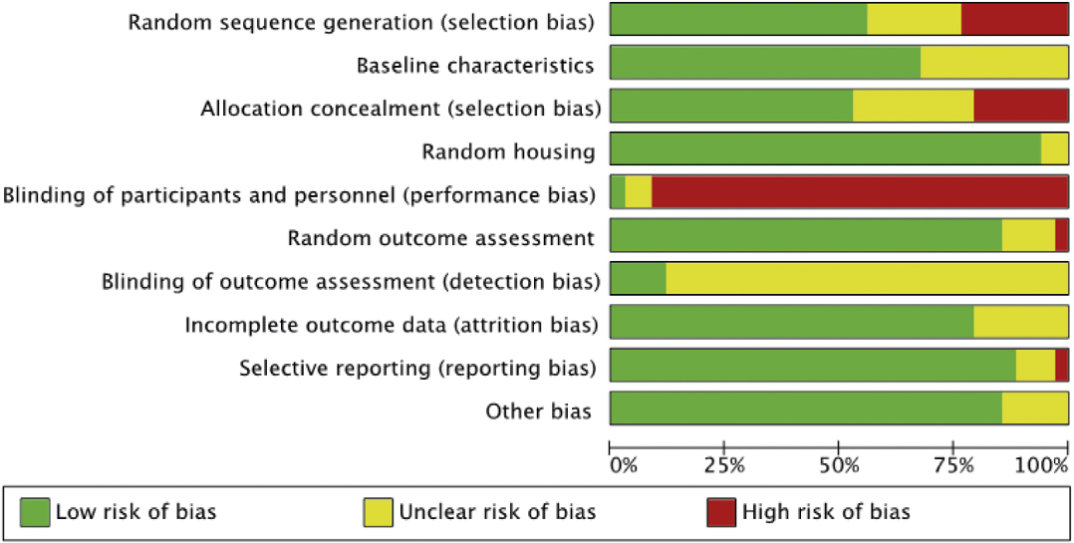
Risk of bias assessment graph for a sample of 34 papers included in the systematic review of physical enrichment in indoor cattle.

The quality of included studies was assessed through SYRCLE’s Risk of Bias tool (Hooijmans *et al.* 2014). The tool includes a set of criteria for assessing the risk of bias, including selection bias, performance bias, detection bias, attrition bias, reporting bias, and other sources of bias. Selection bias was evaluated by analysing the methods of sequence generation, comparing baseline characteristics, and reviewing allocation concealment techniques — procedures that prevent foreknowledge of group assignments during the enrolment of participants. Performance bias involved recording the allocation methods of animals to treatment groups and assessing whether the care providers and researchers were blinded to treatment conditions. Detection bias was examined by verifying if the selection of animals for outcome analysis was randomised and whether outcome assessments were conducted using random methods. Also, we assessed whether the included studies maintained blinding of outcome assessors to the intervention each animal received to minimise detection bias. We evaluated whether all animals initially included in the studies were accounted for in the result data to prevent attrition bias. It was checked whether there was a lack of selective outcome reporting by determining whether the included studies excluded any predefined outcomes or failed to report certain data. For each domain, the risk of bias was rated as low, unclear, or high. Table 2 shows a summary of the evaluation results for each study in the review and Figure 2 illustrates the risk of bias on a domain basis.

**Table 2.**
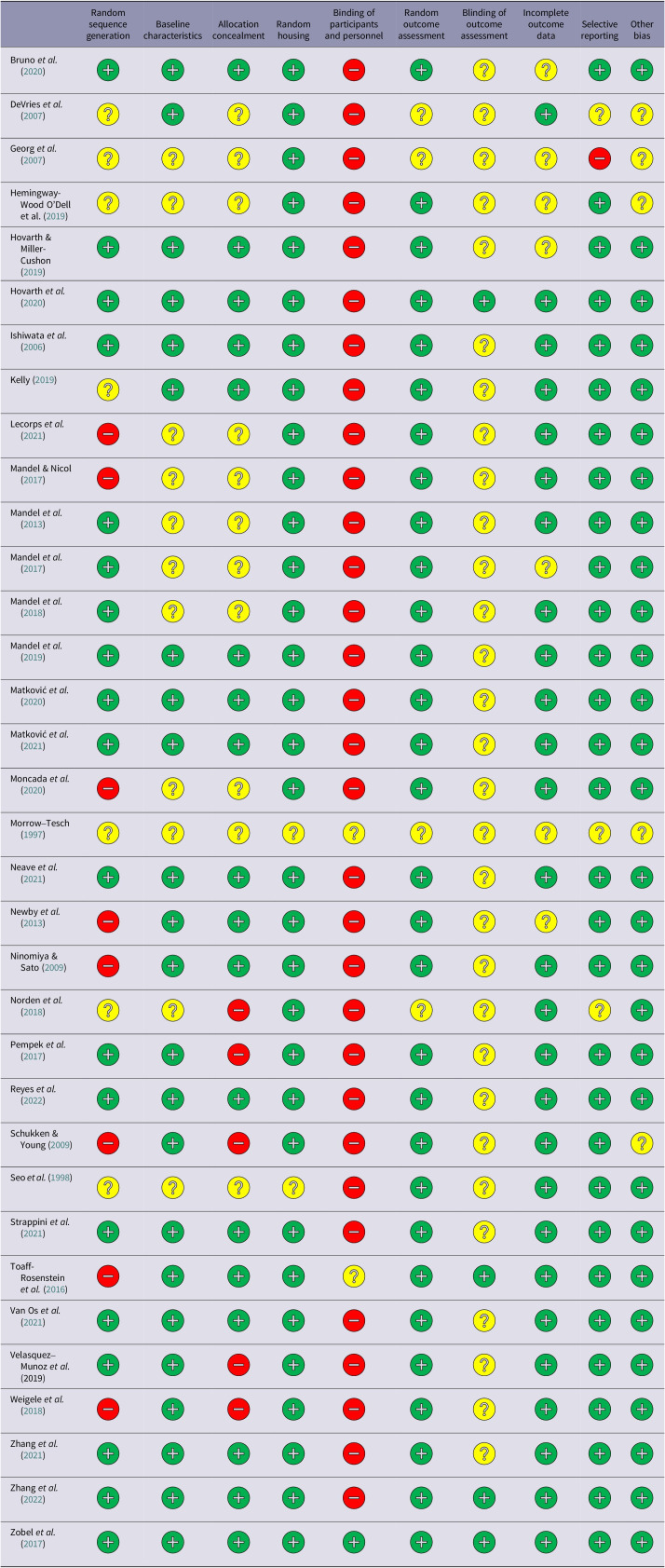
SYRCLE’s risk of bias assessment for studies included in the systematic review of physical environmental enrichment to improve animal welfare-related outcomes in indoor cattle. Each potential source of bias is indicated on the X-axis, with the studies listed on the Y-axis. (+: low risk, ?:unclear risk, -: high risk of bias)

### Data extraction

Data from the included studies are shown in Table 3 (treatment-control trials) and Table 4 (observational studies): study design, study population, type of physical environmental enrichment, measured welfare outcomes, and direction of effect. In this systematic literature review, both treatment-control trials and observational studies without a control group have been employed. Treatment-control trials are listed in Table 3. Conversely, observational studies without control groups, due to their unique methodological and analytical prerequisites, are shown in Table 4. The separation of these two types of studies has enabled us to gain a comprehensive understanding of the various factors influencing the outcomes and simultaneously respect the distinctive features and contributions of each study type to our research.

**Table 3.** Characteristics of included treatment-control trials in the systematic review of physical environmental enrichment in indoor cattle husbandry

Study	Age	Enrichment items	Number of Animals	Outcome	Direction of Effect
(Hemingway-Wood O’Dell *et al.* 2019)	Calf	teat rope	24	Starter intake Cross-sucking behaviour Play behaviour Total nutritive behaviour Time spent eating straw	= ↓ ↑ = =
(Horvath *et al.* 2020)	Calf	Brush	20	Usage of item Feeding time Cleanliness score Pen-directed sucking Grooming Non-nutritive oral behaviour	↑ ↑ ↑ ↓ = ↓
(Horvath & Miller-Cushon 2019)	Calf	Brush	32	Usage of items Cleanliness score Self-grooming Allogrooming	↑ = ↑ =
(Morrow-Tesch 1997)	Calf	Kong toys Chain Plastic ball Calf lolly Braden bottle	18	Usage of items Cross-sucking Standing Lying	↑ ↓ = =
(Neave *et al.* 2021)	Calf	Brush Manila rope	64	Usage of items Play behaviour Lying Grooming	↑ = = ↑
(Ninomiya & Sato 2009)	Calf	Brush Log Wooden board	20	Agonistic behaviours Investigative behaviours Social behaviours	↓ = =
(Norden *et al.* 2018)	Calf	Brush Bottle	12	Usage of items Feed conversion	↑ =
(Pempek *et al.* 2017)	Calf	Brush Teat Lolly Chain	19	Usage of items Sucking pen Locomotor play Growth Starter intake	↑ = ↑ = =
(Seo *et al.* 1998)	Calf	Teat	16	Tongue playing Grooming	= =
(Velasquez-Munoz *et al.* 2019)	Calf	Brush	162	Active time Eating Ruminating Growth	= ↑ = =
(Zhang *et al.* 2022)	Calf	Brush Chain Teat Hanging hay-net	46	Growth rate Exploratory behaviour Cross-sucking Agonistic behaviour	↑ ↑ = =
(Zhang *et al.* 2021)	Calf	Brush Chain Teat	47	Hay intake Ruminating Growth rate Play behaviour Tongue rolling Cross-sucking Allogrooming	↑ = ↑ = = ↓ =
(Kelly 2019)	Heifer	Brush Ball	24	Usage of items Aggression Play behaviours Rumination Growth Eating Lying Standing	↑ = ↑ = = = ↑ ↓
(Matkovic *et al.* 2020)	Heifer	Brush	66	Usage of items Aggression Social behaviours Hairless spots Cleanliness	↑ = = = ↑
(Matkovic *et al.* 2021)	Heifer	Brush	66	Salivary cortisol concentration	=
(Van Os *et al.* 2021)	Heifer	Brush	52	Usage of items Grooming	↑ ↑
(DeVries *et al.* 2007)	Cattle	Brush	144	Usage of item	↑
(Ishiwata *et al.* 2006)	Cattle	Drum-can with plastic turf	48	Usage of item Inactive behaviour Standing Eating Grooming Agonistic behaviours Investigative behaviours Tongue rolling Dopamine concentration Carcase belly fat	↑ = ↑ ↓ = = ↑ = ↑ ↑
(Mandel *et al.* 2019)	Cattle	Brush	36	Hearth rate Exploratory behaviours Locomotion Vocalisation	= ↑ = =
(Newby *et al.* 2013)	Cattle	Brush	16	Usage of item Grooming	↑ ↑
(Schukken & Young 2009)	Cattle	Brush	400	Usage of items Clinical mastitis incidence	↑ ↓

Calf = younger than one-year age, Heifer = adult cattle who has not produced 1 calf, Cattle = adult cattle who has produced at least 1 calf/male adult cattle, EE = environmental enriched, C = control (no enrichment), “↑” = increased with respect to comparator, “↓” = decreased with respect to comparator, “=” = equivalent to comparator

**Table 4.** Characteristics of observational studies in the systematic review of physical environmental enrichment in indoor cattle husbandry

Study	Age	Enrichment items	Number of Animals	Outcome
(Georg 2007)	Calf	Brush Ball	72	Usage of items
(Zobel *et al.* 2017)	Calf	Brush Rope	16	Usage of items
(Bruno *et al.* 2020)	Heifer	Jolly Ball Broom head Knot rope Pas-a Fier Roller	20	Usage of items Standing Lying Eating Exploratory behaviour Rumination Dry matter intake
(Reyes *et al.* 2022)	Heifer	Brush	63	Usage of items Competition
(Lecorps *et al.* 2021)	Cattle	Brush	54	Usage of items
(Mandel *et al.* 2018)	Cattle	Brush	209	Usage of items Locomotion score
(Mandel *et al.* 2017)	Cattle	Brush	88	Usage of items
(Mandel *et al.* 2013)	Cattle	Brush	40	Usage of items
(Mandel & Nicol 2017)	Cattle	Brush	136	Usage of items
(Moncada *et al.* 2020)	Cattle	Brush	48	Usage of items
(Strappini *et al.* 2021)	Cattle	Brush Cowhide Rope	25	Usage of items
(Toaff-Rosenstein *et al.* 2016)	Cattle	Brush	40	Number of contacts with brush Time spending feeding
(Weigele *et al.* 2018)	Cattle	Brush	17 farms	Number of visits to brush Lying Eating Ruminating

Calf = younger than one-year age, Heifer = adult cattle who has not produced 1 calf, Cattle = adult cattle who has produced at least 1 calf/male adult cattle

Meta-analyses were conducted on three key outcomes: growth rate, cross-sucking and locomotor play due to the following factors. Firstly, these outcomes were more consistently and accurately reported across the studies compared to other behaviours and measures. Secondly, these outcomes were consistently measured using similar methodological approaches and units, facilitating reliable comparisons. However, there were challenges related to the heterogeneity of the studies, including differences in the breeds of cattle, the types of environmental enrichment used and the housing conditions. The management practices could all potentially influence the outcomes. Despite our focus on these three outcomes for our meta-analyses, we acknowledge the importance of other behaviours and measures explored in the selected studies. These outcomes were examined in the narrative synthesis of our review.

In order to provide a common scale of measurement across studies, effect size was calculated using the standardised mean difference (Cohen’s d). The mean effect size and its 95% confidence interval were calculated for each outcome. The Z-value tested the null hypothesis that the mean effect size is zero. If the *P*-value associated with the Z-value was less than the defined alpha threshold of 0.05, the null hypothesis was rejected. To assess heterogeneity, we employed the Q-statistic, which tests the null hypothesis that all studies share a common effect size. In line with recommendations from Section 10.10.2 of the *Cochrane Handbook* (Higgins & Cochrane Collaboration 2019), we set the alpha threshold at 0.10 for the Q-test. This higher threshold accounts for the Q-test’s low power — particularly in meta-analyses with relatively few studies — and helps detect potentially meaningful heterogeneity. We acknowledge that 0.10 is not universally standard but is supported under certain circumstances to better identify between-study variability. The I^2^ statistic estimated the percentage of total variation across studies that is due to true between-study differences rather than chance. Given the anticipated variability in study designs and populations, a random-effects model was chosen for our meta-analyses. The random-effects model accounts for both within-study and between-study variance, thereby providing a more conservative estimate in the face of heterogeneity among studies. To generate these analyses and effect size calculations, we used the software Comprehensive Meta-Analysis Version 4 (Borenstein *et al.* 2022).

Visual representation of our meta-analysis results was achieved through the creation of forest plots, which display effect sizes and confidence intervals. These plots also enabled us to assess the presence and extent of heterogeneity among studies, as quantified by the I^2^ statistic. This statistic describes the percentage of total variation across studies that is due to heterogeneity rather than chance. Forest plots were created using the Review Manager software version 5.4 (Review Manager 2020).

## Results

In the screening phase of this systematic review, a total of 372 articles were identified. All these articles were written in English. Of these, 76 duplicate records were removed, leaving us with 296 unique articles for screening. The titles and abstracts of these articles were scanned to evaluate their relevance to our research question. Articles not related to the use of physical environmental enrichment in indoor farm management were excluded. This resulted in the exclusion of 202 articles, thereby leaving us with 94 full-text articles for a more detailed review. Upon a more thorough inspection of these 94 articles, several were found not to meet our specific inclusion criteria. Seven articles were excluded because they focused on a type of enrichment other than physical environmental enrichment; 38 were excluded because they addressed a population other than indoor-housed cattle; and 14 were excluded because they were review articles, not original research. Finally, after these exclusions, 33 original research articles and one report were eligible and included in our review for data extraction.

These studies have been divided into two categories based on their study design: treatment-control trials and observational studies. Of the 34 papers, the majority (n = 21) of the studies were treatment-control trials and a smaller number (n = 13) were observational studies. Treatment-control trials were calf-centric with 12 of 21 studies focusing on calves and a further four studies of breeding replacements. A summary of these studies is provided in Table 3. Observational studies often featured older animals, capturing a diverse age range (Table 4). Observational studies generally had larger sample sizes, providing a broader perspective on cattle behaviour across different farms and conditions.

Across all studies (treatment-control and observational), a range of enrichment types for calves and heifers including brush (n = 30), rope (n = 5), teat/lolly (n = 5), chain (n = 4), ball (n = 2), bottle (n = 2), and cowhide/block (n = 3), were evaluated. Only one study of adult cattle included any enrichment other than cattle brushes which were cow hide and ropes.

### Risk of bias assessment report

The risk of bias assessment of the included studies of the systematic review was completed using SYRCLE’s Risk of Bias tool (Hooijmans *et al.* 2014). The assessment revealed that 90% of studies had a high risk of bias in the domain of researcher blinding. This was due to the presence of physical enrichment items in the experimental pens which are visible to observers and prevent blinding. In addition, 30% of the studies had a significant risk of bias in order sequence generation and allocation concealment. Sequence generation and allocation concealment are two components that ensure the random assignment of participants to treatment groups and are important for reducing the risk of selection bias. The higher risk of bias in sequence generation and allocation concealment indicates unclear or inadequate disclosure of the methods used for how subjects are assigned to different groups. Furthermore, 70% of the studies showed that the risk of blinding in outcome assessment was unclear. To avoid outcome assessment bias, inter-rater reliability statistics can be established among outcome assessors. Despite these limitations, random housing, selective outcome reporting, and other forms of bias were generally assessed as low risk, suggesting that these studies were conducted effectively, and their findings are valid.

### Meta-analysis

#### Growth rate

**Figure 3.**
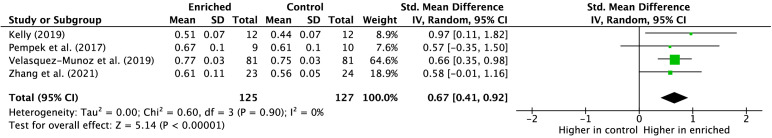
Forest plot of comparison of growth rate between cattle with (enriched) or without (control) environmental enrichment. For each study, the effect size is indicated by a green square, whose size reflects the study’s relative weight in the meta-analysis, with the horizontal line through each square indicating the confidence interval. The black diamond at the bottom represents the pooled estimate and its confidence interval. Values to the right of the y-axis favour the enriched group.

The effect of enrichment items on daily weight gain was analysed based on four studies that met the inclusion criteria (Pempek *et al.* 2017; Kelly 2019; Velasquez-Munoz *et al.* 2019; Zhang *et al.* 2021). Figure 3 showed that the effect of enrichment items on daily weight gain was higher on the right (positive direction) in the enrichment group and higher weight gain on the left (negative direction) in the control group. There was a significant mean effect size of 0.67 kg d^–1^ (Z-value [5.14; *P* < 0.001]). The lack of significant heterogeneity among studies was reflected in the Q-statistic (Q = 0.60, df = 3; *P* = 0.90) and I^2^ (0%). Tau-squared, the variance of true effect sizes, is 0.000 in kg d^–1^. The meta-analysis indicates that enrichment items have a significant effect on daily weight gain, with the enrichment group showing a higher growth rate in the positive direction, resulting in a mean effect size of 0.67.

#### Cross-sucking behaviour

**Figure 4.**
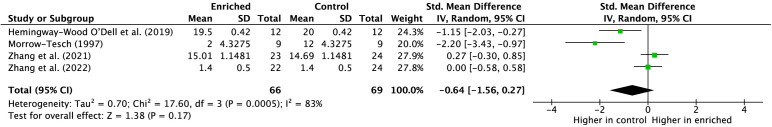
Forest plot comparing cross-sucking behaviour between cattle with (enriched) or without (control) environmental enrichment. Each study is represented by a green square, whose size reflects the study’s relative weight in the meta-analysis, and the horizontal line through each square indicates the confidence interval. The black diamond at the bottom represents the overall pooled effect estimate and its confidence interval. Values to the right of the y-axis favour the enriched group, while values to the left favour the control group.

Four studies were included based on the defined inclusion criteria stated in the *Materials and methods* (Morrow-Tesch 1997; Hemingway-Wood O’Dell 2019; Zhang *et al.* 2021, 2022). The forest plot shows increased cross-sucking behaviour on the right (positive direction) in the enrichment group and increased cross-sucking behaviour on the left (negative direction) in the control group (Figure 4). However, the mean effect size of –0.64 (% of total observed behaviours) (Z-value [1.38; *P* = 0.17]) indicate these effects were not significant. However, the Q-statistic (Q = 17.60, df = 3; *P* = 0.0005) and I^2^ (87%) indicated significant heterogeneity among the study outcomes. Tau-squared, the estimator of the true variance of the underlying effects, is 0.70. Overall, the meta-analysis suggests that enrichment items have an effect on reducing cross-sucking behaviour, the control group showing an increase in cross-sucking behaviour in the negative direction, resulting in a mean effect size of –0.64.

#### Locomotor play

**Figure 5.**
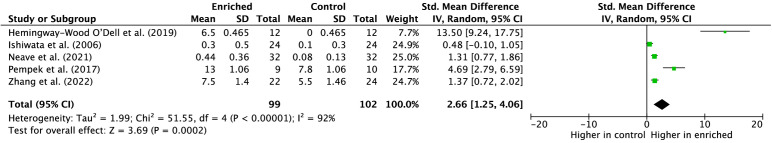
Forest plot comparing locomotor play between cattle with (enriched) or without (control) environmental enrichment. Each study is represented by a green square, whose size reflects the study’s relative weight in the meta-analysis, and the horizontal line through each square indicates the confidence interval. The black diamond at the bottom represents the overall pooled effect estimate and its confidence interval. Values to the right of the y-axis favour the enriched group.

Based on the inclusion criteria, five studies were included to analyse the effect of enrichment items on locomotor play (Ishiwata *et al.* 2006; Pempek *et al.* 2017; Hemingway-Wood O’Dell 2019; Neave *et al.* 2021; Zhang *et al.* 2022). The forest plot indicated that the effect of enrichment items on locomotor play was higher on the right (positive direction) in the enrichment group and higher on the left (negative direction) in the control group (Figure 5). The mean effect size of 2.66 (% of total observed behaviours) (Z-value [3.69; *P* = 0.002]) shows a significant effect of enrichment items on locomotor play. High heterogeneity was found among the studies, as indicated by the Q-statistic (Q = 51.55, df = 4; *P* < 0.00001) and I^2^ (92%). Tau-squared, the variance of true effect sizes, is 1.99 in % of observations. Based on this meta-analysis, the use of enrichment items significantly increases locomotor play.

## Discussion

This systematic review underscores the substantial influence of physical environmental enrichment on the behaviour of indoor-housed cattle, affirming its importance for promoting natural behaviours and, consequently, enhancing animal welfare. The enrichment devices assessed, including brushes, manila ropes, teat/lolly toys, chains, balls, and bottles, have been shown to facilitate essential activities such as grooming, foraging, and exploration, which are vital for the cattle’s psychological and physical health. Notably, grooming behaviour can be effectively stimulated by using brushes (Horvath & Miller-Cushon 2019).

A closer examination of the 34 studies included in this review reveals a predominant use of controlled trials (n = 21) compared to observational studies (n = 13), indicating a stronger focus on experimental approaches in this field. The controlled trials predominantly featured younger cattle, specifically calves (n = 12) and breeding replacements (n = 4), as detailed in Table 3. This focus on younger animals could be because they are subject to a number of challenges during these early stages of development which have the potential to negatively affect growth. It could also relate to the practicalities of replicated study design being significantly more challenging with adult dairy cattle due to space and management constraints. Conversely, observational trials usually featured adult dairy cattle (Table 3). Retaining these observational trials in our review provides a more comprehensive view of cattle behaviour across different farms and conditions.

The enrichment types deployed were varied; however, brushes were overwhelmingly the most common form of enrichment (n = 30), suggesting they are a standard tool in the industry. Other enrichments like ropes (n = 5), teat/lolly toys (n = 5), chains (n = 4), balls (n = 2), bottles (n = 2), and cowhide/blocks (n = 3) were less frequently employed. It is noteworthy that enrichment for adult cattle was rarely beyond the provision of brushes, with only one study incorporating alternative enrichments such as cowhide and ropes. This is likely to be because of the actual and perceived constraints of adult cattle accommodation by both researchers and farmers, e.g. cubicle sheds and frequent slurry scraping of passageways which can limit the fixing of enrichment devices.

Our analysis critically considers the positive and negative effects of the use of physical enrichment devices on production-related, health-related and behavioural outcomes with the aim of providing evidence to support effective enrichment strategies for cattle farming systems.

### Production-related outcomes

#### Growth rate and feed intake

The meta-analysis of growth rate outcomes (Pempek *et al.* 2017; Kelly 2019; Velasquez-Munoz *et al.* 2019; Zhang *et al.* 2021) revealed a significant increase in growth rate (0.67 kg d^–1^) for calves exposed to environmental enrichment compared with those without enrichments. One explanation for this could be that spending additional time undertaking activities directed at the enrichment devices lowers stress responses which are known to have an energy cost to the individual animal (Harris 2015) leading to the partitioning of energy from the consumed ration to the immune system before animal growth. Enrichment items, especially those that encourage activity, oral exploration and grooming, can support various behavioural needs (Mkwanazi *et al.* 2019). It is possible that these items not only fulfil the calves’ natural tendencies but also indirectly foster behaviours like social learning (Harland & Dalrymple-Alford 2020; Zentall 2021). This result also dispels any concerns of livestock producers, that when calves are engaged with enriching items or activities, they may spend less time feeding and consequently grow more slowly. The energy density of diets fed to ruminants in confined environments are typically greater than at pasture, allowing these animals to consume feed more quickly than when grazing outdoors (McGrath *et al.* 2018), leaving sufficient time for non-food related activities such as engagement with enrichment and social behaviours.

### Behavioural outcomes

#### Cross-sucking behaviour

The systematic review examined how enrichment devices, such as brushes, chains and teats affect cross-sucking behaviour in calves by analysing data from five relevant studies (Morrow-Tesch 1997; Pempek *et al.* 2017; Hemingway-Wood O’Dell 2019; Zhang *et al.* 2021, 2022). The meta-analysis indicates that these devices, brushes and teats do not significantly decrease cross-sucking behaviour. The mean effect size is 0.64. Although the meta-analysis included only four studies with varied types of enrichment, pooling these studies was appropriate as all enrichment types aimed to mitigate cross-sucking behaviour by providing alternative stimuli or resources. The drivers for cross-sucking behaviour are not fully understood but it may be influenced by various factors such as social dynamics and environmental conditions, housing design, space allowance (Jensen 2003). For instance, Zhang *et al.* (2022) discovered that social housing arrangements like pair housing could worsen sucking tendencies, although this was compared with single-housed calves with no opportunities to cross-suck. When comparing pairs with and without enrichment, they found that the presence of enrichment items reduced cross-sucking behaviour in pair-housed calves. This implies that while social housing arrangements influence behaviour, the inclusion of physical enrichment can mitigate some of the negative effects. Although the meta-analysis does not provide evidence for a significant reduction in cross sucking through enrichment strategies, it does highlight a negative trend in control groups, without such interventions. This contradiction emphasises the importance of implementing nuanced enrichment strategies tailored to address behavioural needs of cattle within each unique farm setting.

#### Locomotor play and exploratory behaviour

Our meta-analysis reviewed findings from five studies (Ishiwata *et al.* 2006; Pempek *et al.* 2017; Hemingway-Wood O’Dell 2019; Neave *et al.* 2021; Zhang *et al.* 2022) that quantitatively assessed the influence of environmental enrichment on locomotor play in dairy calves. No studies involving adult cows were included, as our search did not return any quantitative analyses for this age group. The results indicate a positive effect with an effect size of 2.66 (*P* = 0.0002). This demonstrates that enrichment has an influence on locomotor play. Despite variations among the included studies, enrichment consistently increases locomotor play, which is crucial for both physical and cognitive development in calves (Uren 2018). Enrichment items, like brushes, toys and drum-cans not only enhance play but also fulfil behavioural needs of calves diverting them from engaging in less desirable activities (Zhang *et al.* 2021). Specifically, the meta-analysis included the use of brushes, which consistently enhanced play behaviour and grooming activities across several studies. Manila ropes encouraged exploratory actions and foraging behaviour, while teats and chains provided opportunities for oral engagement and reduced cross-sucking behaviour (Morrow-Tesch 1997; Hemingway-Wood O’Dell 2019; Neave *et al.* 2021). Balls and toys increased overall play behaviour, and drum-cans with plastic turf contributed to varied sensory and physical stimulation (Ishiwata *et al.* 2006; Kelly 2019). The evidence emphasises the importance of tailoring the use of enrichment for calves based on their ages and the design of the enrichment items. While certain enrichments, such as brushes and balls, consistently enhance play behaviour, others like manila ropes and teat toys encourage more specific behaviours such as exploratory actions, foraging, and oral engagement, thereby creating a diverse and stimulating environment for the calves. Studies have shown that providing multiple enrichment objects, such as mechanical brushes and ropes, can add complexity and novelty to barren environments, leading to increased usage by the calves (Strappini *et al.* 2021; Zhang *et al.* 2021). Inclusion of enrichment devices promoting locomotor play therefore has the potential to move animals from ‘a life worth living’ to ‘a good life’, one with opportunities for positive experiences such as pleasure (Edgar *et al.* 2013).

#### Aggression and stereotypic behaviours

The literature review process also highlighted that aggression and stereotypic behaviours were influenced by enrichment in livestock (Table 3). For example, providing brushes as an enrichment correlated with lying and social behaviours among Japanese black and Shorthorn calves as well as being shown by Ninomiya (2019) to lead to a reduction in negative behaviours such as excessive licking, biting at the bars of enclosures, or pacing, which are often indicative of stress or frustration. Interestingly, Matkovic *et al.* (2020) noted that enriched environments reduced aggressive behaviours in cattle housed at higher stocking densities, whereas no significant effect was observed in cattle housed at lower densities. This suggests that enrichment effectiveness can vary with herd density. Enrichment objects like drum-cans significantly redirected steer behaviour towards active behaviours such as eating (Ishiwata *et al.* 2006) suggesting that enrichment positively channels their energy. Although a small number of studies, these strategies appear to alleviate frustration and potentially reduce behaviours by encouraging natural behaviours and minimising competition over resources (Shepherdson 2003).

### Health-related outcomes

#### Cleanliness score

Environmental enrichment tools like brushes can significantly impact cleanliness scores in cattle, which are important indicators of animal welfare as a result of their effects on health and comfort (Hedman *et al.* 2021). The Welfare Quality® Network emphasises that cleanliness is a key aspect of animal welfare, as it is linked to the prevention of diseases and the overall well-being of livestock (Welfare Quality® 2009). Brushes were one of the most common enrichment provisions and improved cleanliness has been reported for adult cows, heifers and calves (Tables 3 and 4). Horvath and Miller-Cushon (2019) noted that the use of brushes as enrichment tools was associated with a significant improvement in cleanliness scores. The implementation of brushes effectively promotes self-grooming, enhances hygiene, and reduces disease incidence, thereby potentially improving health and comfort for dairy cattle (Ninomiya 2019). However, another study highlighted that stocking density can be a limiting factor for the availability of such enrichment tools. It was found that higher stocking densities can limit access to these tools, resulting in decreased frequency of their use and diminishing their benefits on cleanliness scores (Matkovic *et al.* 2020). Beyond the direct hygienic benefits, there is a suggestion that the act of brushing may serve a pleasurable function for cattle, contributing to an enhanced state of well-being (Miranda *et al.* 2023). It shows that cattle may engage with brushes out of an intrinsic desire for pleasure, indicating a behaviour that goes beyond their well-being to include aspects of happiness and fulfilment. While the primary goal of brush use aligns with health and hygiene, these additional advantages linked to the happiness of cows deserves greater exploration to determine if enrichment items such as brushes can provide animals with positive welfare experiences.

#### Lying

Lying behaviour in cattle, a vital indicator of welfare related to rest and rumination (Tucker *et al.* 2021), has been shown to be influenced by environmental enrichment. In one study, brushes, logs, and wooden walls positively affected lying times, particularly in Japanese black calves (Ninomiya & Sato 2009). However, these results are not consistent with Ishiwata *et al.* (2006) who used drum-cans as enrichment and reported higher instances of resting and rumination activities in pens without enrichment compared to with, suggesting that there might be other factors influencing these behaviours, for example, space allocation and pen design, also critically shape these behaviours. Divergence is also seen in the effectiveness of different enrichment types. For instance, brushes have been associated with increased lying times as opposed to other enrichments like hanging balls (Kelly 2019), suggesting the importance of enrichment compatibility with cattle preferences. In light of these findings, it is crucial that enrichment strategies are carefully designed to align with natural cattle behaviours and preferences to truly enhance welfare and to avoid inadvertently reducing other desirable behaviours.

#### Interaction between health status and life stage and enrichment

The use of mechanical brushes as environmental enrichment has been positively correlated with welfare in dairy cattle (McConnachie *et al.* 2018). However, the interaction of cows with these devices varies depending on their health status and life events. Studies have demonstrated that cows tend to increase their activity levels when using brushes, with healthy cows showing more significant increases compared to unhealthy cows (Toaff-Rosenstein *et al.* 2016; Mandel *et al.* 2017). Post-calving, cows with access to brushes are more driven towards calf-licking behaviours, beneficial for both hygiene and calf health (Newby *et al.* 2013). The use of brushes is subject to variation with significant life events such as calving or separation from calves often resulting in decreased brush interaction (Lecorps *et al.* 2021); a crucial consideration as it underscores the importance of tailoring enrichment to fit changing behavioural needs. Health conditions like mange similarly deter brush usage, though normal activity levels often resume post-treatment (Moncada *et al.* 2020). The strategic placement of brushes — away from feeding areas — also appears to enhance their utilisation, particularly among healthy compared to lame cows (Mandel *et al.* 2018). However, the design of cubicle sheds for adult cows often presents constraints that may limit the optimal placement of such enrichment tools. These structures need to be thoughtfully designed not only to provide comfort and ease of access to feed and water but also to incorporate elements like brushes in locations that maximise voluntary use without disrupting normal traffic patterns or feeding behaviour. This consideration is essential to ensure that enrichment tools are accessible and beneficial to all cows, regardless of their physical location within the shed. This pattern suggests that while brushes generally contribute to welfare, their use is modulated by the cow’s health and environment. As such, the design and implementation of enrichment strategies must account for ensuring these dynamics are truly effective. Future research should focus upon understanding the connection between cattle health, behaviour, and enrichment usage, aiming to develop optimised approaches for welfare improvement.

#### Impact of enrichment on physiological stress indicators

The assessment of physiological stress indicators can be used to indicate the well-being of cattle and the efficacy of environmental enrichment. Matkovic *et al.* (2021) suggests that grooming brushes and salt blocks correlate with lower cortisol levels in heifers, especially during the initial fattening period, indicating a stress-reducing benefit of enrichment. Additionally, the use of a drum-can as enrichment was linked to higher dopamine levels in beef cattle, showing not just enhanced engagement but also an improved emotional state with potential positive impacts on health and productivity (Ishiwata *et al.* 2006). Similarly, heifers at lower stocking density environments without enrichment exhibited higher cortisol levels compared to those in enriched lower density environments, indicating an increase in stress-related behaviours (Matkovic *et al.* 2021). Yet, the introduction of grooming tools did not reduce other stress indicators such as heart rate in isolated dairy cows (Mandel *et al.* 2019), suggesting that enrichment may not be sufficient to overcome certain stressful situations. The implications are multifaceted: stress, known to influence feed intake and growth (Blokhuis *et al.* 1998), can be mitigated through strategic environmental enrichment. These findings highlight the necessity of adopting enrichment that resonates with the physiological and psychological needs of cattle to enhance their welfare and productivity.

## Animal *welfare implications*

This systematic review and meta-analysis highlight existing research on environmental enrichment for the welfare of cattle. Items like brushes, chains and teats can effectively reduce behaviours such as cross-sucking while promoting actions like play, grooming and feeding. By encouraging positive behaviours and decreasing stress-related activities, these items create an engaging environment and can improve animal welfare, crucial in indoor settings where natural behaviours may be limited. There is also some evidence presented for enhancements in growth rates and overall productivity highlighting further advantages of integrating enrichment into cattle farming.

The review also points out research gaps especially concerning the effects of enrichment items other than brushes on adult dairy cows. There is a necessity for studies that explore the intricate relationships between enrichment usage, surroundings and individual characteristics of animals such as age group and health conditions. Addressing these gaps will be vital in developing widely applicable enrichment programmes.

Enrichment tools can improve the well-being of cattle and contribute to a quality of life. This review suggests that there is now adequate evidence to recommended that future welfare programmes and guidelines incorporate environmental enrichment for cattle.

## Conclusion

This systematic review and meta-analysis have provided an insight into the diverse impacts of environmental enrichment on cattle at all life stages, showcasing a general trend towards improved welfare. Enrichment items like brushes, chains, and teats have been particularly effective in reducing problematic behaviours such as cross-sucking, with varying degrees of success depending on the type and implementation of the enrichment. The review process has highlighted a growing body of research demonstrating a range of positive benefits associated with the use of physical enrichment items, including alleviating problem behaviours, encouraging positive behaviours like play and allogrooming, and increasing growth and productivity. Enrichment items have the potential to enhance cattle welfare and promote a good life and should therefore be included in future welfare schemes and codes of practice. However, the review also identified a number of important gaps in the research, such as the limited number of studies on the impacts of enrichments other than brushes in adult dairy cows. It also highlights the complex interactions between enrichment use, environment, individual animal life stage, and health status, which can contribute to the conflicting results observed for certain outcomes.
